# Self-Vibration of a Liquid Crystal Elastomer Fiber-Cantilever System under Steady Illumination

**DOI:** 10.3390/polym15163397

**Published:** 2023-08-13

**Authors:** Kai Li, Yufeng Liu, Yuntong Dai, Yong Yu

**Affiliations:** School of Civil Engineering, Anhui Jianzhu University, Hefei 230601, China

**Keywords:** self-vibration, liquid crystal elastomer, light-powered, fiber-cantilever

## Abstract

A new type of self-oscillating system has been developed with the potential to expand its applications in fields such as biomedical engineering, advanced robotics, rescue operations, and military industries. This system is capable of sustaining its own motion by absorbing energy from the stable external environment without the need for an additional controller. The existing self-sustained oscillatory systems are relatively complex in structure and difficult to fabricate and control, thus limited in their implementation in practical and complex scenarios. In this paper, we creatively propose a novel light-powered liquid crystal elastomer (LCE) fiber-cantilever system that can perform self-sustained oscillation under steady illumination. Considering the well-established LCE dynamic model, beam theory, and deflection formula, the control equations for the self-oscillating system are derived to theoretically study the dynamics of self-vibration. The LCE fiber-cantilever system under steady illumination is found to exhibit two motion regimes, namely, the static and self-vibration regimes. The positive work done by the tension of the light-powered LCE fiber provides some compensation against the structural resistance from cantilever and the air damping. In addition, the influences of system parameters on self-vibration amplitude and frequency are also studied. The newly constructed light-powered LCE fiber-cantilever system in this paper has a simple structure, easy assembly/disassembly, easy preparation, and strong expandability as a one-dimensional fiber-based system. It is expected to meet the application requirements of practical complex scenarios and has important application value in fields such as autonomous robots, energy harvesters, autonomous separators, sensors, mechanical logic devices, and biomimetic design.

## 1. Introduction

Self-excited oscillation refers to a recurring oscillatory phenomenon that arises from external steady excitations. Conventional mechanical oscillation is usually subjected to periodic external stimulus that generates periodic forced motion in time and space. In contrast to forced oscillation, self-oscillation can actively adjust its own motion, provide feedback in response to steady external stimulus, and obtain regular energy to maintain its periodic motion [1,2,3,4]. Self-oscillation can not only obtain energy directly and independently from the external environment to maintain its own motion mode, but also its vibration frequency and amplitude depend only on the inherent parameters of the structure. It does not require other complex controllers to achieve periodic oscillation [5,6], so from the perspective of dynamics theory, self-oscillation is of great significance for understanding new behaviors such as bifurcation, chaos, synchronization, and other non-equilibrium dynamics in nonlinear systems. It is a typical non-equilibrium dynamical process in nonlinear systems [7]. Self-oscillating systems have broad application prospects and revolutionary impact on autonomous robots [8,9,10,11,12], energy harvesters [13,14], independent separators, sensors [15], mechanical logic devices [16], and biomimetic design.

In recent years, active materials such as hydrogels [17,18], dielectric elastomers [19], ion gels [20], and thermally responsive polymer materials [21] have exhibited different responses under different stimulus conditions. These responses generally change the morphology and motion state of the active materials themselves. People have established various self-oscillating systems and multiple self-sustained motion modes using the properties of active materials, including bending [22,23,24], swimming [25], swinging [26], rolling [2,9,10,27], rotating [28,29], twisting [30,31], vibration [6], and even synchronized motion of several coupled self-oscillators [32,33]. In general, in all dynamic systems, there is energy dissipation [34], and in practical environments, the vibrations tend to approach an equilibrium state. Therefore, designing different types of self-oscillating systems is a challenging process. In a constant environment, how to enable the system to absorb energy autonomously, compensate for the damping dissipation, and maintain periodic motion is the key to realize self-oscillation. A large number of self-excited oscillatory systems have been established based on various feedback mechanisms. These different feedback mechanisms typically lead to different self-sustained motion modes, such as self-shadowing [35,36,37], coupling of liquid evaporation and membrane deformation [38], coupling mechanism of air expansion and liquid column motion [39], and coupling of plate bending and chemical reaction [40], all of which can cause self-excited oscillations.

The advantages of light in various stimuli are its sustainability, accuracy, controllability [41,42], and non-contact. Optically-responsive materials that can convert near-infrared and visible light into thermal energy, such as carbon nanotubes, graphene, and liquid crystal elastomers (LCEs) [43,44,45,46,47,48] have good photomechanical effects [49,50,51,52,53,54]. Among them, LCEs are important optically responsive materials, synthesized from anisotropic rod-shaped liquid crystal molecules and stretchable long-chain polymers. When liquid crystal monomers are subjected to external stimuli such as light, heat, electricity, and magnetism, they will rotate or undergo phase transitions, thereby changing their configuration and generating macroscopic deformation [55,56]. LCEs typically offer advantages of large deformation, fast deformation response, recoverable deformation, low noise, easy remote control, and easy manipulation. Based on LCEs, photomechanical effects have been utilized to build various self-sustained oscillatory systems, including but not limited to shuttling [57], bending [58], rotation [29,30,55], spinning [59], curling [60], oscillating [61,62], buckling [63,64,65], rolling [28], floating [66], twisting [67], vibration [68], swimming [25], chaos [69], and even several synchronous motions coupled with self-excited oscillations [2,27,34]. These LCE-based self-sustained oscillatory systems have attracted much attention in both fundamental and applied research [55,70,71,72].

Although a large number of self-sustained oscillatory systems have been constructed, these systems generally have complex structures, are difficult to manufacture and control, and may not meet the requirements of complex practical applications. In this article, we propose a novel and simple LCE fiber-cantilever system that exhibits self-sustained oscillation under steady illumination and essentially functions as a “self-shadowing” system. Compared to previous self-oscillating systems such as balls [66] and tubes [42], the structure of one-dimensional fiber is relatively simple, making it easy to assemble and disassemble. It should also be noted that the proposed LCE fiber-cantilever system may exhibit a dependence on the angle of illumination in practice. Furthermore, the system is highly extensible, holding potential for constructing more complex LCE fiber-based systems to achieve advanced self-sustained motions. The objective of this research is to build the LCE fiber-cantilever system and investigate its self-oscillation characteristics under stable illumination. Meanwhile, we discuss the underlying mechanisms of self-oscillation and systematically explore the impacts of various physical and geometric parameters on the system’s amplitude and frequency.

The organization of this paper is as follows. First, in Section 2, considering the dynamic LCE model and beam theory, the theoretical model and control equations for the LCE fiber-cantilever system are established. Then, in Section 3, two motion regimes of the LCE fiber-cantilever system are obtained by numerical calculations, and the mechanism of its self-vibration is explained in detail. Next, in Section 4, the influences of various system parameters on the amplitude and frequency of self-vibration are discussed in detail. Finally, the results are summarized.

## 2. Theoretical Model and Formulation

In this section, we first propose a light-powered self-oscillation system containing an LCE fiber, an oblique bending cantilever, and a mass block. Then, we present a theoretical model for the self-oscillation system based on the dynamic LCE model [8] and beam theory [73]. The dynamic control equations of the system, the evolution law of the *cis* number fraction in LCE, and the nondimensionalization of the system parameters are then given in turn.

### 2.1. Dynamics of System

**Figure 1:**
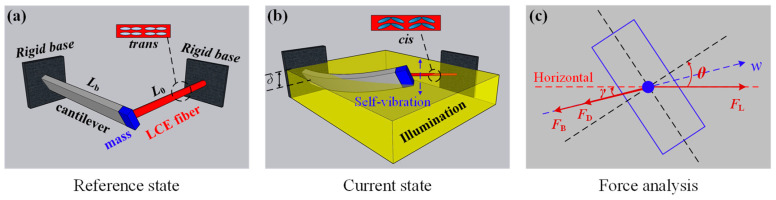
Schematic of an LCE fiber-cantilever system containing an LCE fiber, a lightweight cantilever beam, and a mass block: (**a**) Reference state; (**b**) Current state; (**c**) Force analysis. $F_{L}$ denotes the tension of the LCE fiber, $F_{B}$ represents the force exerted by the beam on the mass block, $F_{D}$ represents the air damping force, γ is the angle between the cantilever deflection and the horizontal direction, and θ is the inclined angle of cantilever.

Figure 1 schematically describes the proposed LCE fiber-cantilever system, in which an LCE fiber, a lightweight cantilever beam and a mass block are included. The lightweight cantilever of length $L_{B}$ at an angle θ from the horizontal, is fixed on a vertical rigid base. The mass block with mass m at the cantilever end is connected by the LCE fiber fixed on another vertical rigid base to form a tension string system. The bending effect of gravity on the cantilever can be ignored as it is much smaller than other forces. Both the torsion and displacement of the cantilever along the length are small, so the mass block is assumed to move in a plane. We take the initial position of the mass block as the origin of the coordinate system and establish the coordinate axis along the direction of cantilever deflection. The initial length of LCE fiber is $L_{0}$. In addition, the masses of the LCE fiber and the cantilever are much less than the mass m, so they are neglected.

The system is placed under steady illumination as shown in Figure 1b, with the yellow region representing the illumination zone with a height of δ. Generally, chromophores in the LCE fiber upon illumination undergo series of *trans-cis-trans* isomerization cycles ending up in the change of the orientation of the *trans-isomer* long axis [74]. In case of non-polarized light illumination, the long axes orient towards the illumination direction, while in case of illumination with polarized light, the long axes orient perpendicular to the light polarization, because of the direction-dependent absorption of the chromophore. These changes can change the order parameter of the LCE and lead in some geometries to contraction of the fiber. As the LCE fiber contracts, the cantilever bends further into the dark zone. When the LCE fiber is in the dark, the azobenzene molecules in it switch from *cis* to *trans*, causing the light-driven contraction of the LCE fiber to recover. Subsequently, the tension of the LCE fiber decreases andthe cantilever returns to the illumination zone due to the structural resistance. Through the proper adjustment of the system parameters and initial conditions, the LCE fiber-cantilever system can maintain continuous self-oscillation.

The mass block is subjected to the tension of LCE fiber, the structural resistance form cantilever, and the air damping force, as depicted in Figure 1c. In the deflection direction, the control equation for the nonlinear dynamics model of mass block can be expressed as follows:

(1) $$m\ddot{w}=F_{L}\cdot \cos\gamma -F_{B}-F_{D}$$

where $\ddot{w}$ refers to the acceleration of the mass block, $F_{L}$ denotes the tension of the LCE fiber, $F_{B}$ represents the force exerted by the beam on the mass block, $F_{D}$ represents the air damping force, and γ is the angle between the cantilever deflection and the horizontal direction.

Through the beam deflection theory, the moment of inertia formula, and the trigonometric function, it can be calculated $\gamma =\arctan[r^{2}\tan\theta ]-\theta$, where r refers to the ratio of cantilever height to width.

The tension of LCE fiber is related to its elongation and cross-sectional area, which can be described as

(2) $$F_{L}=\frac{E_{L}A_{L}\cdot \Delta L}{L}=\frac{E_{L}A_{L}\{[L_{0}+2w(t)\cdot \cos\gamma ]-L_{0}[1+\varepsilon_{L}(t)]\}}{L_{0}[1+\varepsilon_{L}(t)]}$$

where $F_{L}$ refers to the elastic modulus of the LCE fiber, $A_{L}$ refers to the cross-sectional area of the LCE fiber, $L_{0}$ is the original length of LCE fiber, w(t) represents the cantilever-end deflection, i.e., the displacement of the mass block, and $\varepsilon_{L}(t)$ represents the light-driven contraction strain of LCE fiber.

It is assumed that the cantilever beam is always in a state of small deformation, while the theory of linear elasticity is applied, thus the structural resistance from cantilever is proportional to the displacement, that is

(3) $$F_{B}=\frac{3E_{B}I_{B}}{L_{B}^{3}}\cdot w(t)$$

where $L_{B}$ is the cantilever length, $E_{B}I_{B}$ is the bending stiffness of the cantilever.

The damping force is assumed to be linearly proportional to the velocity of the mass block, with the formula being

(4) $$F_{D}=\beta \cdot \dot{w}(t)$$

where β denotes the air damping coefficient and $\dot{w}$ is the velocity of the mass block.

Thus far, substituting Equations (2)–(4) into Equation (1), we have

(5) $$m\frac{d^{2}w(t)}{dt^{2}}=E_{L}A_{L}\cdot \cos\gamma \cdot \frac{\left\{[L_{0}+2w(t)\cdot \cos\gamma ]-L_{0}[1+\varepsilon_{L}(t)]\right\}}{L_{0}[1+\varepsilon_{L}(t)]}.$$

### 2.2. Dynamic LCE Model

This section mainly describes the dynamic model of the light-driven contraction in LCE fiber. The fiber radius is assumed to be much smaller than the penetration depth of light, and no absorption gradient within the fiber is considered. The LCE fiber-cantilever system uses a linear model, which is adopted to describe the relationship between the *cis* number fraction φ(t) in LCE and the light-driven contraction of LCE, that is

(6) $$\varepsilon_{L}=-C_{0}\cdot \varphi (t)$$

where $C_{0}$ is the contraction coefficient.

The light-driven contraction in LCE depend on the cis number fraction φ(t) [75,76]. The study by Yu et al. found that the *trans-to-cis* isomerization of LCE could be induced by UV or laser with wavelength less than 400 nm [77]. In this study, a ‘push-pull’ mechanism is considered to calculate the *cis* number fraction [76]. The number fraction φ(t) of the *cis-isomer* depends on the thermal excitation from *trans* to *cis*, the thermally driven relaxation from *cis* to *trans*, and the light driven relaxation from *trans* to *cis*. Supposing that the thermal excitation from *trans* to *cis* can be ignored, the governing equation for the evolution of the *cis* number fraction can be formulated as

(7) $$\frac{\partial \varphi }{\partial t}=\eta_{0}I_{0}(1-\varphi )-\frac{\varphi }{T_{0}}$$

where $T_{0}$ refers to the thermally driven relaxation time from the *cis* to *trans*, $I_{0}$ denotes the light intensity, and $\eta_{0}$ is the light absorption constant. By solving Equation (7), the *cis* number fraction can be described as

(8) $$\varphi (t)=\frac{\eta_{0}T_{0}I_{0}}{\eta_{0}T_{0}I_{0}+1}+(\varphi_{0}-\frac{\eta_{0}T_{0}I_{0}}{\eta_{0}T_{0}I_{0}+1})\exp[-\frac{t}{T_{0}}(\eta_{0}T_{0}I_{0}+1)]$$

where $\varphi_{0}$ represents the initial *cis* number fraction at t=0.

In illuminated state, for initially zero-number fraction, i.e., $\varphi_{0}=0$, Equation (8) can be simplified as

(9) $$\varphi (t)=\frac{\eta_{0}T_{0}I_{0}}{\eta_{0}T_{0}I_{0}+1}\{1-\exp[-\frac{t}{T_{0}}(\eta_{0}T_{0}I_{0}+1)]\}$$

In non-illuminated state, namely $I_{0}=0$, Equation (8) can be simplified as

(10) $$\varphi (t)=\varphi_{0}\exp(-\frac{t}{T_{0}})$$

where the undetermined $\varphi_{0}$ can be set to be the maximum value of φ(t) in Equation (9), namely, $\varphi_{0}=\frac{\eta_{0}T_{0}I_{0}}{\eta_{0}T_{0}I_{0}+1}$. Then Equation (10) can be rewritten as

(11) $$\varphi (t)=\frac{\eta_{0}T_{0}I_{0}}{\eta_{0}T_{0}I_{0}+1}\exp(-\frac{t}{T_{0}})$$

### 2.3. Nondimensionalization

We introduce the following dimensionless quantities by defining: $\bar{w}=\frac{w}{L_{0}}$, initial velocity ${\bar{\dot{w}}}_{0}=\frac{T_{0}{\dot{w}}_{0}}{L_{0}}$, $\bar{t}=\frac{t}{T_{0}}$, spring constant ${\bar{K}}_{L}=\frac{E_{L}AT_{0}^{2}}{mL_{0}}$, flexural stiffness ${\bar{K}}_{B}=\frac{3E_{B}I_{B}T_{0}^{2}}{mL_{B}^{3}}$, $\bar{\beta }=\frac{\beta T_{0}}{m}$, ${\bar{I}}_{0}=\eta_{0}T_{0}I_{0}$, $\bar{\delta }=\frac{\delta }{L_{0}}$, and $\bar{\varphi }=\frac{\varphi (\eta_{0}T_{0}I_{0}+1)}{\eta_{0}T_{0}I_{0}}$, to simplify the governing equations Equations (5) and (9)–(11).

The dimensionless form of Equation (5) can be expressed as

(12) $$\bar{\ddot{w}}(\bar{t})={\bar{K}}_{L}\cdot \cos\gamma \cdot [\frac{1}{1-C_{0}\cdot \bar{\varphi }(\bar{t})}+\frac{\bar{w}(\bar{t})\cdot \cos\gamma }{1-C_{0}\cdot \bar{\varphi }(\bar{t})}-1]-{\bar{K}}_{B}\cdot \bar{w}(\bar{t})-\bar{\beta }\cdot \bar{\dot{w}}(\bar{t})$$

In illuminated state, Equation (9) can be rewritten as

(13) $$\bar{\varphi }=1-\exp[-\bar{t}({\bar{I}}_{0}+1)]$$

and in non-illuminated state, Equation (11) becomes

(14) $$\bar{\varphi }=\exp(-\bar{t})$$

Equations (12)–(14) are utilized to regulate the self-vibration of the LCE fiber-cantilever system in the presence of steady illumination. These equations involve a time-varying fractional quantity associated with the cis isomer and closely linked to the light intensity. To solve these intricate linear equations, the fourth-order Runge–Kutta method is employed for numerical computations using the Matlab software. Moreover, Equations (13) and (14) are employed to determine the cis number fraction φ and time length $\bar{t}$, enabling the calculation of tension $F_{L}$, air damping force $F_{D}$, and structural resistance $F_{B}$ of the LCE fiber. By iterating calculation with given parameters ${\bar{\dot{w}}}_{0}$, ${\bar{K}}_{L}$, ${\bar{K}}_{B}$, $\bar{\beta }$, ${\bar{I}}_{0}$, $C_{0}$, θ, r, and $\bar{\delta }$, the dynamics of the LCE fiber-cantilever system can be obtained.

## 3. Two Motion Regimes and Mechanism of Self-Vibration

In this section, through solving the control equation Equation (12), we first propose two typical motion regimes of the LCE fiber-cantilever system, which are distinguished as static regime and self-vibration regime. Next, the corresponding mechanism of self-vibration is elaborated in detail.

### 3.1. Two Motion Regimes

In order to further study the self-vibration behavior of the LCE fiber-cantilever system, we first need to determine the typical values for the dimensionless system parameters. Based on the existing experiments and information [78,79,80], Table 1 gathers the typical values of the system parameters required in current paper. The corresponding dimensionless parameters are listed in Table 2. In the following section, these values of parameters are used to study the self-vibration of the LCE fiber-cantilever system under steady illumination. It is worth noting that the small deformation hypothesis can be verified under these given parameters.

**Figure 2:**
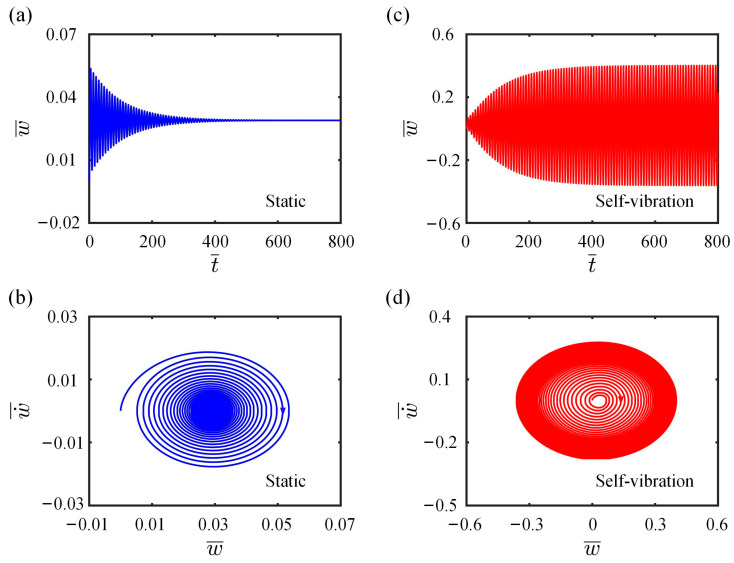
Two typical motion regimes of the LCE fiber-cantilever system under steady illumination: static regime and self-vibration regime. (**a**) Time-history curve of the displacement with ${\bar{I}}_{0}=0.25$; (**b**) Phase trajectory diagram with ${\bar{I}}_{0}=0.25$; (**c**) Time-history curve of the displacement with ${\bar{I}}_{0}=0.5$ and (**d**) Phase trajectory diagram with ${\bar{I}}_{0}=0.5$.

By solving Equations (12)–(14), the time histories and phase trajectories for the LCE fiber-cantilever system can be obtained, with examples for ${\bar{I}}_{0}=0.25$ and ${\bar{I}}_{0}=0.5$ shown in Figure 2. The other parameters used in the calculation are set as $C_{0}=0.25$, ${\bar{K}}_{L}=0.2$, ${\bar{K}}_{B}=0.7$, $\bar{\beta }=0.02$, ${\bar{\dot{w}}}_{0}=0$, $\bar{\delta }=0.03$, r=2 and $\theta =\frac{\pi }{4}$. In Figure 2a,b, the amplitude of the cantilever-end deflection gradually decreases with time due to the damping dissipation, and the system eventually reaches a stationary position at equilibrium, which is referred to as the static regime. In contrast, Figure 2c,d show that the system initially vibrates from a static equilibrium position and then progressively increases in vibration amplitude over time until it remains constant. On exposure to steady illumination, the LCE fiber-cantilever system eventually presents a continuous periodic vibration, which we refer to as the self-vibration regime.

### 3.2. Mechanism of the Self-Vibration

**Figure 3:**
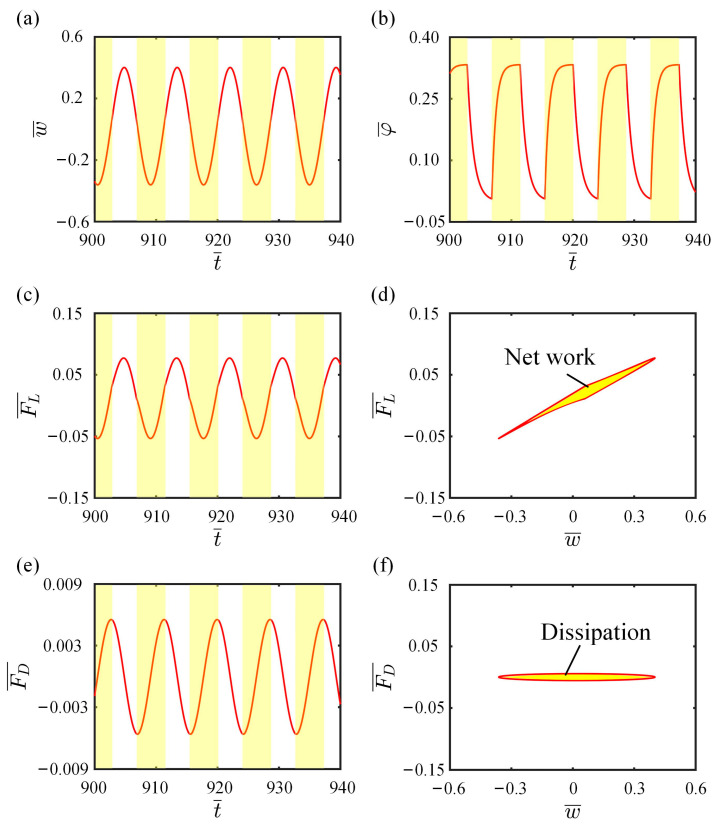
Self-vibration mechanism of the LCE fiber-cantilever system (**a**) Time-history curve of the cantilever-end deflection. (**b**) Time variation of the light-driven contraction of LCE fiber. (**c**) Time variation of the tension of LCE fiber. (**d**) Dependence of the tension of LCE fiber on the cantilever-end deflection. (**e**) Time variation of the damping force. (**f**) Dependence of the damping force on the cantilever-end deflection.

**Figure 4:**
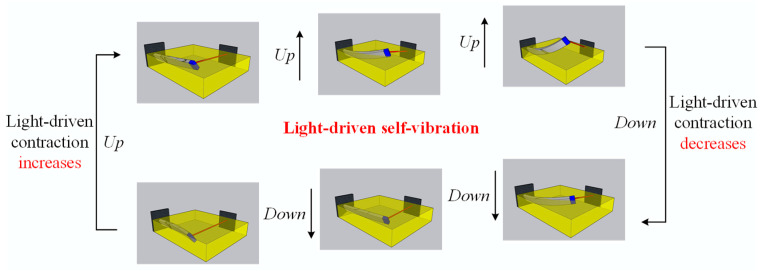
Snapshots of the LCE fiber-cantilever system in one cycle during the self-vibration. Under steady illumination, the system exhibits a continuous periodic self-vibration due to the periodic variation of light-driven contraction.

In this section, the mechanism of self-vibration will be explained in detail. To better understand the energy compensation mechanism of the LCE fiber-cantilever system, we plot the relationship curves for some key physical quantities in the self-vibration process. In this case, the system parameters are selected as ${\bar{I}}_{0}=0.5$, $C_{0}=0.25$, ${\bar{K}}_{L}=0.25$, ${\bar{K}}_{B}=0.7$, $\bar{\beta }=0.02$, ${\bar{\dot{w}}}_{0}=0$, $\bar{\delta }=0.03$, r=2, and $\theta =\frac{\pi }{4}$. Figure 3a illustrates the cantilever-end deflection over time, with the yellow area indicating that the LCE fiber is in the illumination zone. As the system vibrates continuously, the LCE fiber also oscillates back and forth between the illumination and dark zones, and the change in the *cis* number fraction $\bar{\varphi }$ over time is drawn in Figure 3b. It is clearly observed that as the illumination condition changes, the *cis* number fraction changes rapidly at first and then slowly approaches a critical value determined by the contraction coefficient $C_{0}$. In addition, Figure 4 illustrates several characteristic snapshots for the self-vibration of the LCE fiber-cantilever system during one cycle under steady illumination.

Figure 3c presents the periodic time variation of the tension of the LCE fiber. The tension decreases first and then increases in the illumination zone, while the opposite is true in the dark zone. The hysteresis loop shown in Figure 3d indicates that the LCE fiber-cantilever system maintains its oscillation as the LCE fiber absorbs light energy and does work. The area enclosed by the loop represents the net work done by the tension of the LCE fiber in one cycle, with a value of approximately 0.0029. Like the tension of the LCE fiber, it is clear from Figure 3e that the damping force also presents a periodic time variation. Figure 3f plots the dependence of the damping force on the cantilever-end deflection, which also forms a closed loop representing the damping dissipation, with a value being calculated to be about 0.0029. The net work done by the tension of LCE fiber is exactly equal to the damping dissipation, implying that the energy consumed by the system motion is compensated by the light energy absorbed by the LCE fiber, thus maintaining the self-vibration.

## 4. Parametric Study

In the mechanical model of the self-vibration for the LCE fiber-cantilever system described above, there are nine dimensionless system parameters: ${\bar{I}}_{0}$, $C_{0}$, ${\bar{K}}_{L}$, ${\bar{K}}_{B}$, $\bar{\beta }$, ${\bar{\dot{w}}}_{0}$, $\bar{\delta }$, r, and θ. In this section, we investigate in detail the effects of these system parameters on the self-vibration of the LCE fiber-cantilever system, including its frequency and amplitude. The dimensionless self-vibration frequency and amplitude are denoted by f and A, respectively.

### 4.1. Effect of Light Intensity

**Figure 5:**
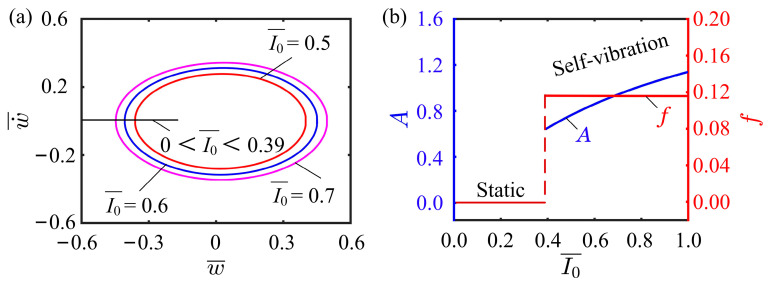
Effect of light intensity on the self-vibration. (**a**) Limit cycles with ${\bar{I}}_{0}=0.5$, ${\bar{I}}_{0}=0.6$ and ${\bar{I}}_{0}=0.7$. (**b**) Variations of amplitude and frequency with different light intensities.

The effect of light intensity on the self-vibration is discussed in current subsection. In this case, the values of the other parameters are, $C_{0}=0.25$, ${\bar{K}}_{L}=0.25$, ${\bar{K}}_{B}=0.7$, $\bar{\beta }=0.02$, ${\bar{\dot{w}}}_{0}=0$, $\bar{\delta }=0.03$, r=2, and $\theta =\frac{\pi }{4}$. The limit cycles of the self-vibration are depicted in Figure 5a, where ${\bar{I}}_{0}=0.39$ is the critical value of light intensity between the static and self-vibration regimes. When the light intensity is below 0.39, the system is in static regime, while above 0.39, the system is in self-vibration regime. When the light intensity is relatively small, the LCE fiber does not absorb enough light energy to offset the damping dissipation, thus it cannot maintain its continuous motion and comes to rest. Conversely, when the light intensity is large enough, the light energy absorbed by the system can compensate for the damping dissipation, so as to maintain its own motion. Figure 5b describes the effect of light intensity on the self-vibration amplitude and frequency. With the increasing light intensity, the amplitude increases, while the frequency remains essentially constant. Larger light intensity allows the system to absorb more light energy, thereby maintaining oscillation with higher amplitude. These results suggest that increasing the light intensity is crucial for improving the energy utilization efficiency of the LCE fiber-cantilever system.

### 4.2. Effect of Contraction Coefficient

**Figure 6:**
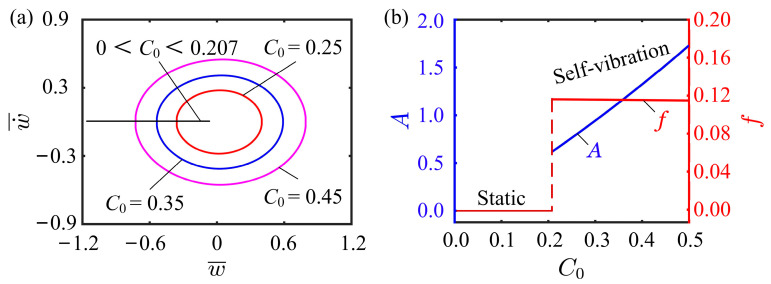
Effect of contraction coefficient on the self-vibration. (**a**) Limit cycles with $C_{0}=0.25$, $C_{0}=0.35$, and $C_{0}=0.45$. (**b**) Variations of amplitude and frequency with different contraction coefficients.

This subsection mainly discusses the effect of contraction coefficient on the self-vibration. Here, the values of the other parameters are ${\bar{I}}_{0}=0.5$, ${\bar{K}}_{L}=0.25$, ${\bar{K}}_{B}=0.7$, $\bar{\beta }=0.02$, ${\bar{\dot{w}}}_{0}=0$, $\bar{\delta }=0.03$, r=2, and $\theta =\frac{\pi }{4}$. Figure 6a plots the limit cycles for different contraction coefficients. Obviously, there exists a critical value for contraction coefficient to trigger the self-vibration, which is numerically determined to be 0.207. A small contraction coefficient means a low light energy input, and there is not enough energy to compensate for the damping dissipation. For $C_{0}=0.25$, $C_{0}=0.35$, and $C_{0}=0.45$, the self-vibration can be triggered. Figure 6b presents the dependencies of the self-vibration amplitude and frequency on the contraction coefficient. The larger the contraction coefficient, the higher the amplitude. As the contraction coefficient increases, the LCE fiber makes more efficient use of the illumination, absorbs more light energy, and shifts the system from a static regime to a self-vibration regime, resulting in an increase in the amplitude. The result implies that increasing the contraction coefficient of LCE material can improve the efficient conversion of light energy to mechanical energy.

### 4.3. Effect of Spring Constant

**Figure 7:**
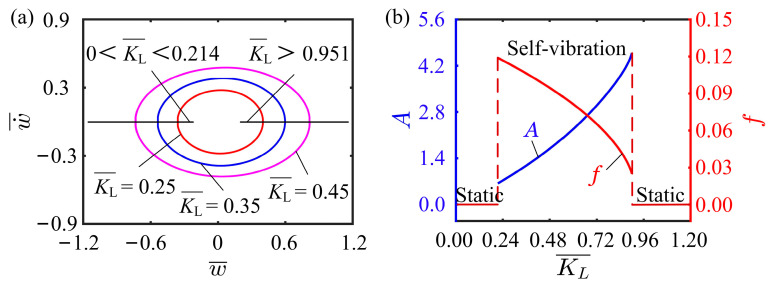
Effect of spring constant on the self-vibration. (**a**) Limit cycles with ${\bar{K}}_{L}=0.25$, ${\bar{K}}_{L}=0.35$, and ${\bar{K}}_{L}=0.45$. (**b**) Variations of amplitude and frequency with different spring constants.

This subsection mainly focuses on the effect of spring constant on the self-vibration. In this case, the values of the other parameters are ${\bar{I}}_{0}=0.5$, $C_{0}=0.25$, ${\bar{K}}_{B}=0.7$, $\bar{\beta }=0.02$, ${\bar{\dot{w}}}_{0}=0$, $\bar{\delta }=0.03$, r=2, and $\theta =\frac{\pi }{4}$. Figure 7a displays the limit cycles for different spring constants, among which two critical spring constants exist for triggering the self-vibration. It is clear to see that the system is in the static regime when the spring constant is below 0.214 or above 0.951. This can be explained by the relationship between the spring constant and the tension of the LCE fiber. When the spring constant is small, the tension of the LCE fiber is small, which is not enough to force the system to remain in oscillation. When the spring constant is large, the tension of the LCE fiber can be equal to the structural resistance, thus allowing the whole system to equilibrate the forces and reach a static regime. Figure 7b illustrates that the spring constant has a significant effect on the amplitude and frequency of the self-vibration. As the spring constant increases, the amplitude increases, while the frequency decreases. This is because the spring constant determines the driving force of the system, which in turn affects the oscillatory behavior of the system. Therefore, when we design the LCE fiber-cantilever system, the adjustment of the spring constant can be used to control its amplitude and frequency to achieve better performance. For example, in some robotic applications, the LCE fiber-cantilever system is required to realize stable motion or grasp an object, we can select the appropriate spring constant according to the desired motion mode and the weight of the object, so as to keep the system stable and have good accuracy during operation. In addition, when designing suspended structures or other oscillatory systems, the amplitude and frequency can also be controlled according to the variation of the spring constant to achieve better performance.

### 4.4. Effect of Flexural Stiffness

**Figure 8:**
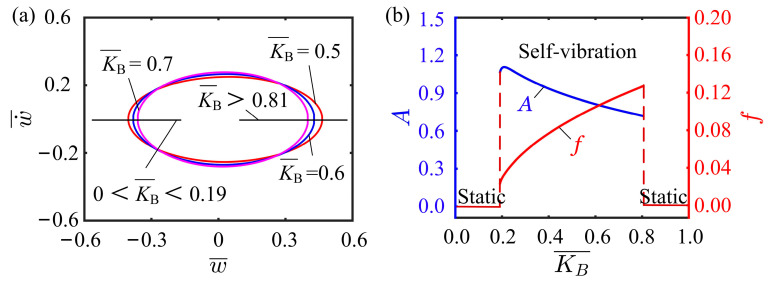
Effect of flexural stiffness on the self-vibration. (**a**) Limit cycles with ${\bar{K}}_{B}=0.5$, ${\bar{K}}_{B}=0.6$, and ${\bar{K}}_{B}=0.7$. (**b**) Variations of amplitude and frequency with different bending stiffnesses.

The influence of flexural stiffness on the self-vibration is provided for ${\bar{I}}_{0}=0.5$, $C_{0}=0.25$, ${\bar{K}}_{L}=0.25$, $\bar{\beta }=0.02$, ${\bar{\dot{w}}}_{0}=0$, $\bar{\delta }=0.03$, r=2, and $\theta =\frac{\pi }{4}$. The limit cycles for different flexural stiffnesses are drawn in Figure 8a. The flexural stiffness has two critical values for the transition between the static and self-vibration regimes, which are numerically calculated to be around 0.19 and 0.81. When the flexural stiffness is small, the structural resistance of the cantilever is small, and the net work done by the tension of the LCE fiber is not sufficient to maintain the self-vibration. When the flexural stiffness is large, the structural resistance from the cantilever is so great that the tension of the LCE fiber cannot drive the system to oscillate. Figure 8b plots the variations of self-vibration amplitude and frequency with different flexural stiffnesses. As the flexural stiffness increases, the amplitude decreases, while the frequency increases. This can be explained by the beam theory, where the greater the flexural stiffness of the beam, the greater the recovery force on the beam, thus preventing further bending of the beam. As a result, the amplitude decreases. Therefore, to improve the system stability, it is a good way to choose the appropriate flexural stiffness of the beam.

### 4.5. Effect of Damping Coefficient

**Figure 9:**
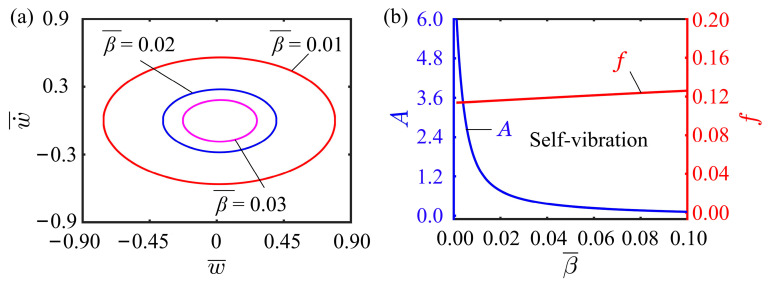
Effect of damping coefficient on the self-vibration. (**a**) Limit cycles with $\bar{\beta }=0.01$, $\bar{\beta }=0.02$, and $\bar{\beta }=0.03$. (**b**) Variations of amplitude and frequency with different damping coefficients.

Figure 9 presents the influence of damping coefficient on the self-vibration, with parameters ${\bar{I}}_{0}=0.5$, $C_{0}=0.25$, ${\bar{K}}_{L}=0.25$, ${\bar{K}}_{B}=0.7$, ${\bar{\dot{w}}}_{0}=0$, $\bar{\delta }=0.03$, r=2, and $\theta =\frac{\pi }{4}$. The limit cycles for different damping coefficients can be observed in Figure 9a. It is not difficult to find that the variation of damping coefficient does not affect the motion regime of the LCE fiber-cantilever system. For different damping coefficients, the system is always in a self-vibration regime. The dependencies of the self-vibration amplitude and frequency on the damping coefficient are depicted in Figure 9b. With the increase of damping coefficient, the amplitude decreases sharply and then slowly, presenting the characteristics of an exponential function. In contrast, changes in the damping coefficient have little effect on the frequency. This suggests that the damping coefficient plays an important role in influencing the amplitude and energy level of self-vibration systems. Proper adjustment of the damping coefficient can control the vibration amplitude and energy level of the system to ensure the system stability. Moreover, as the damping coefficient has little effect on the frequency, the damping coefficient and frequency need to be considered comprehensively during the system design process to obtain the optimal scheme. These research results not only provide important application value in the field of engineering design and manufacture, but also provide new ideas and methods for the in-depth understanding of complex systems.

### 4.6. Effect of Initial Velocity

**Figure 10:**
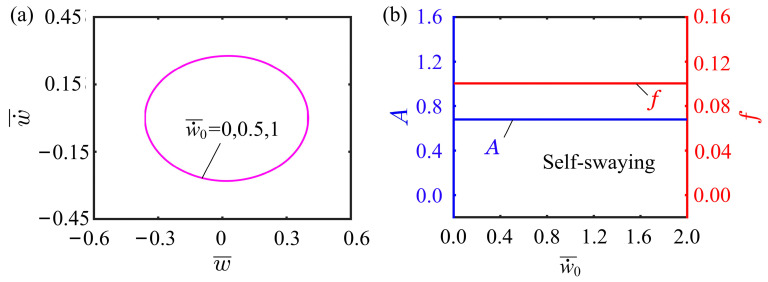
Effect of initial velocity on the self-vibration. (**a**) Limit cycles with ${\bar{\dot{w}}}_{0}=0$, ${\bar{\dot{w}}}_{0}=0.5$, and ${\bar{\dot{w}}}_{0}=1$. (**b**) Variations of amplitude and frequency with different initial velocities.

The effect of initial velocity ${\bar{\dot{w}}}_{0}$ on the self-vibration is displayed in Figure 10, with other parameters being ${\bar{I}}_{0}=0.5$, $C_{0}=0.25$, ${\bar{K}}_{L}=0.25$, ${\bar{K}}_{B}=0.7$, $\bar{\beta }=0.02$, $\bar{\delta }=0.03$, r=2, and $\theta =\frac{\pi }{4}$. ${\bar{\dot{w}}}_{0}=0$, ${\bar{\dot{w}}}_{0}=0.5$, and ${\bar{\dot{w}}}_{0}=1$ are found to successfully trigger the self-vibration, and the limit cycles are plotted in Figure 10a. It is worth mentioning that the limit cycles for these three initial velocities overlap. As can be seen in Figure 10b, the variation of the initial velocity does not affect the amplitude and frequency of the system. Since the self-vibration results from the energy conversion between the damping dissipation and the network done by the tension of the LCE fiber, the self-vibration amplitude and frequency are determined by the internal properties of the system, which is consistent with other self-vibration systems. The initial velocity therefore has no effect on the final amplitude of the system.

### 4.7. Effect of Illumination Zone Height

**Figure 11:**
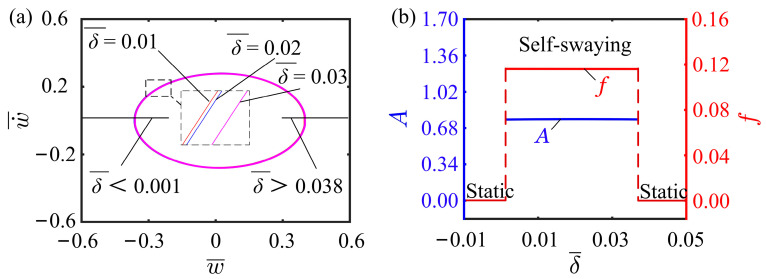
Effect of illumination zone height on the self-vibration. (**a**) Limit cycles with $\bar{\delta }=0.01$, $\bar{\delta }=0.02$, and $\bar{\delta }=0.03$. (**b**) Variations of amplitude and frequency with different illumination zone heights.

This subsection presents a discussion on the effect of illumination zone height on the self-vibration. In the calculation, we set other parameters as ${\bar{I}}_{0}=0.5$, $C_{0}=0.25$, ${\bar{K}}_{L}=0.25$, ${\bar{K}}_{B}=0.7$, $\bar{\beta }=0.02$, ${\bar{\dot{w}}}_{0}=0$, r=2, and $\theta =\frac{\pi }{4}$.As observed from Figure 11a, for the phase transition between the static and self-vibration regimes, two critical illumination zone heights exist with values of 0.001 and 0.037, respectively. When the illumination zone height is less than 0.001 or greater than 0.037, the system is in astatic regime. When the illumination zone height is within the interval of 0.001 and 0.037, the system is in a self-vibration regime. The effect of illumination zone height on the amplitude and frequency is shown in the Figure 11b. Obviously, the amplitude and frequency do not vary with increasing the illumination zone height. This is contributed to the fact that as the illumination zone expands, the tension of the LCE fiber increases, and the structural resistance from cantilever also increases accordingly. Consequently, the system encounters greater resistance during self-vibration, resulting in a drop in amplitude. In conclusion, adjusting the appropriate range of the illumination zone can be more effective in improving the efficiency of light utilization.

### 4.8. Effect of Ratio of Cantilever Height to Width

**Figure 12:**
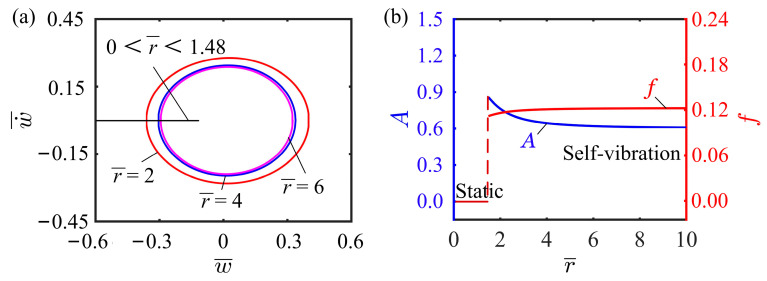
Effect of ratio of cantilever height to width on the self-vibration. (**a**) Limit cycles with r=2, r=4, and r=6. (**b**) Variations of amplitude and frequency with different ratios of cantilever height to width.

This subsection mainly discusses how the ratio of cantilever height to width affects the self-vibration. In this case, the other dimensionless parameters are selected as ${\bar{I}}_{0}=0.5$, $C_{0}=0.25$, ${\bar{K}}_{L}=0.25$, ${\bar{K}}_{B}=0.7$, $\bar{\beta }=0.02$, ${\bar{\dot{w}}}_{0}=0$, $\bar{\delta }=0.03$, and $\theta =\frac{\pi }{4}$. Figure 12a shows the three limit cycles for ratios of cantilever height to width of r=2, r=4, and r=6. The system is in the static regime when the ratio is below 1.48, while it is in the self-vibration regime when the ratio exceeds 1.48. This is due to the small deflection angle of the cantilever end when the ratio of cantilever height to width is small. The longitudinal deflection of the cantilever end is too small for the system to leave the illumination zone, so the system becomes static. Figure 12b depicts how the ratio of cantilever height to width affects the self-vibration amplitude and frequency. As the ratio of cantilever height to width increases, the self-vibration amplitude will first decrease rapidly, and then a marginal effect occurs, slowing down the reduction rate. At the same time, the self-vibration frequency will first increase rapidly, and then a marginal effect appears, slowing down its increase. These findings underscore the significance of meticulous selection of the ratio of cantilever height to width and suggest that opting for an appropriate ratio can effectively enhance the efficiency of converting light energy into mechanical energy.

### 4.9. Effect of Inclined Angle of Cantilever

**Figure 13:**
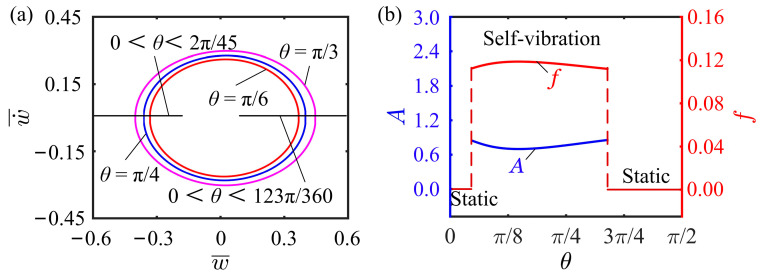
Effect of inclined angle of cantilever on the self-vibration. (**a**) Limit cycles with $\theta =\frac{\pi }{6}$, $\theta =\frac{\pi }{4}$, and $\theta =\frac{\pi }{3}$. (**b**) Variations of amplitude and frequency with different inclined angles of cantilever.

The inclined angle of cantilever affecting the self-vibration is investigated in this subsection, where the other dimensionless parameters are chosen as ${\bar{I}}_{0}=0.5$, $C_{0}=0.25$, ${\bar{K}}_{L}=0.25$, ${\bar{K}}_{B}=0.7$, $\bar{\beta }=0.02$, ${\bar{\dot{w}}}_{0}=0$, $\bar{\delta }=0.03$, and r=2. Figure 13a illustrates the limit cycles for different inclined angles, in which $\theta =\frac{2\pi }{45}$ and $\theta =\frac{123\pi }{360}$ are the two critical inclined angles for the phase transition between the static and the self-vibration regimes. The self-vibration can be triggered with inclined angles of $\theta =\frac{\pi }{6}$, $\theta =\frac{\pi }{4}$, and $\theta =\frac{\pi }{3}$, while the system is in the static regime with $\theta <\frac{2\pi }{45}$ and $\theta >\frac{123\pi }{360}$. Clearly observed from Figure 13b that as the inclined angle of cantilever increases, the self-vibration frequency first increases and then decreases, and conversely the amplitude first decreases and then increases, indicating that there is an optimal inclined angle for the self-excited oscillation. In summary, setting an appropriate inclined angle of cantilever can promote the self-vibration. Too large- or too small- inclined angle of cantilever is not conducive to the self-vibration of the system.

## 5. Conclusions

Self-excited oscillatory systems can maintain continuous motion by absorbing energy from the stable external environment, and possess potential applications in biomedicine, advanced robotics, rescue operations, military industry, and other fields. In order to overcome the disadvantages of existing self-sustained oscillatory systems that are relatively complex in structure and difficult to fabricate and control, we creatively propose a novel light-powered LCE fiber-cantilever system composed of an LCE fiber, a lightweight cantilever beam, and a mass block under steady illumination. The dynamic control equations for the LCE fiber-cantilever system are derived based on the established LCE dynamic model, beam theory, and deflection formula. The solutions of the nonlinear control equations are obtained using the Runge–Kutta numerical calculation method with MATLAB software. The results show that the LCE fiber-cantilever system evolves into two motion regimes, namely the static and self-vibration regimes. We have described these two motion regimes specifically and also revealed the energy compensation mechanism of the system. In a constant illumination, the positive work done by the tension of the LCE fiber is used to compensate for the structural resistance from the cantilever and the air damping, resulting in the contraction and relaxation.

Further numerical calculations show that the light intensity, contraction coefficient, spring constant, flexural stiffness, damping coefficient, ratio of cantilever height to width, and the inclined angle of the cantilever have a considerable effect on the self-vibration amplitude of the system. The spring constant of the LCE fiber and the flexural stiffness of the cantilever beam significantly affect the self-vibration frequency of the system. The illumination zone height has little effect on the amplitude and frequency, and the amplitude and frequency are not affected by the initial velocity. The LCE fiber-cantilever system constructed in this paper is a simple, easy-to-assemble and disassemble, easy-to-prepare, and highly expandable one-dimensional fiber-based system. It is expected to meet the application requirements of practical complex scenarios and has important application value in the fields of autonomous robotics, energy harvesters, autonomous separators, sensors, mechanical logic devices, and bionic design.

## Figures and Tables

**Table 1:** Material properties and geometric parameters.

Parameter	Definition	Value	Unit
$I_{0}$	Light intensity	0~10	kW/m^2^
$C_{0}$	Contraction coefficient	0~0.5	/
$K_{L}$	Spring constant	0.1~1	N/m
$K_{B}$	Flexural stiffness	0.3~3	N/m
β	Damping coefficient	0~0.001	kg/s
$w_{0}$	Initial velocity	0~0.5	mm/s
δ	Height of illumination zone	0~0.1	m
r	Ratio of cantilever height to width	1~20	/
θ	Inclined angle of cantilever	0~1.2	rad
$T_{0}$	Cis- to trans- thermal relaxation time	1~100	ms
$\eta_{0}$	Light-absorption constant	0.001	m^2^/(s∙W)

**Table 2:** Dimensionless parameters.

**Parameter**	${\bar{I}}_{0}$	$C_{0}$	${\bar{K}}_{L}$	${\bar{K}}_{B}$	$\bar{\beta }$	${\bar{\dot{w}}}_{0}$	$\bar{\delta }$	r	θ
**Value**	0~1	0~0.5	0~1.2	0~1	0~0.2	0~5	0~0.1	1~20	0~$\frac{\pi }{2}$

## Data Availability

Not applicable.
